# ELAC2, an Enzyme for tRNA Maturation, Plays a Role in the Cleavage of a Mature tRNA to Produce a tRNA-Derived RNA Fragment During Respiratory Syncytial Virus Infection

**DOI:** 10.3389/fmolb.2020.609732

**Published:** 2021-02-02

**Authors:** Eun-Jin Choi, Wenzhe Wu, Ke Zhang, Inhan Lee, In-Hoo Kim, Yong Sun Lee, Xiaoyong Bao

**Affiliations:** ^1^ Department of Pediatrics, The University of Texas Medical Branch, Galveston, TX, United States; ^2^ Department of Chemistry, The University of Houston Clear Lake, Clear Lake, TX, United States; ^3^ miRcore, Ann Arbor, MI, United States; ^4^ Department of Cancer Biomedical Science, Graduate School of Cancer Science and Policy, National Cancer Center, Goyang, Korea; ^5^ Sealy Center for Molecular Medicine, The University of Texas Medical Branch, Galveston, TX, United States; ^6^ The Institute of Translational Sciences, The University of Texas Medical Branch, Galveston, TX, United States; ^7^ The Institute for Human Infections and Immunity, The University of Texas Medical Branch, Galveston, TX, United States

**Keywords:** RSV, tRF, biogenesis, ELAC2, viral replication

## Abstract

Respiratory syncytial virus (RSV) is the most common cause of lower respiratory tract infection in young children. However, effective treatment against RSV is unavailable. tRNA-derived RNA fragments (tRFs) are a recently discovered family of non-coding RNAs. We made an early observation that RSV infection causes significant induction of tRFs, which are mainly derived from the 5’-end of mature tRNAs (tRF5). However, their functions and biogenesis mechanism are not fully understood. Herein, we identified an enzyme responsible for the induction of a functional tRF5 derived from tRNA-Gln-CTG (tRF5-GlnCTG). We found that tRF5-GlnCTG promotes RSV replication and its induction, assessed by Northern blot and a new qRT-PCR-based method, is regulated by ribonuclease ELAC2. ELAC2-mediated tRF5 induction has never been reported. We also found that ELAC2 is associated with RSV N and NS1 proteins. Given the fact that tRF5-GlnCTG plays a role in RSV replication, the identification of ELAC2 being responsible for tRF5-GlnCTG induction could provide new insights into therapeutic strategy development against RSV infection.

## Introduction

Respiratory syncytial virus (RSV) is the most common cause of lower respiratory tract infections (RTI) in children (Pickles and DeVincenzo, 2015). It is also associated with high morbidity and mortality in at-risk populations including premature infants, the elderly, and immunocompromised patients (Lanari et al., 2004; Falsey et al., 2005; Simon et al., 2007). RSV also contributes significantly to upper RTI resulting in outstanding outpatient clinic visits (Hall et al., 2009). Despite its medical burden, no specific treatments are currently available, suggesting the urgent need to expand our knowledge beyond what we currently know about RSV disease mechanisms, so that novel and critical insights into the development of anti-RSV strategies can be generated.

Non-coding RNAs (ncRNAs) are RNAs, which are not translated into proteins. They include small ncRNAs (sncRNAs, <200 nts) and long ncRNAs (lncRNAs, >200 nts), both of which have emerged as critical regulatory molecules for various physiological and pathological processes including infectious diseases (Palazzo and Lee, 2015; Romano et al., 2017; Chen et al., 2019; Piket et al., 2019). Our recent high-throughput sequencing revealed that the most affected sncRNAs by RSV in airway epithelial cells (AEC) belong to tRNA-derived RNA fragments (tRFs) (Wang et al., 2013). RSV-induced tRFs are not likely degraded byproducts because 1) only a subset of tRNAs produce tRFs; 2) affected tRNAs are cleaved right before their anti-codon loops resulting in 30-nt-long tRFs, which are derived from the 5’-end of corresponding tRNAs (tRF5); and 3) some RSV-induced tRFs are proven to be functional (Wang et al., 2013; Deng et al., 2015; Zhou et al., 2017a). Although the top five induced tRF5s have been experimentally investigated, the functions of other RSV-induced tRFs have not been explored and regulatory molecular mechanisms underlying their induction and activities await the investigation.

To get a big picture of the overall roles of induced tRFs in RSV infection, we continued our study on RSV-induced tRF5-GlnCTG, derived from the 5’-end of mature tRNA decoding GlnCTG. We found that tRF5-GlnCTG facilitates RSV replication. Unlike other tRF5s, whose induction is dominantly controlled by the ribonuclease angiogenin (ANG), the induction of tRF5-GlnCTG is dependent on another ribonuclease ELAC2, the long-form of RNase Z and also an enzyme commonly known for tRNA maturation by removing a 3'-trailer from tRNA precursors (Rossmanith, 2011; Siira et al., 2018) or the induction of tRF-1, the tRF derived from the 3’-end of a tRNA precursor transcript that is not retained in the mature tRNAs (Lee et al., 2009). The role of ELAC2 in cleaving a mature tRNA has never been reported. In addition, RSV N and NS1 may contribute to the biogenesis of tRF5-GlnCTG as they interact with ELAC2 and suppress the luciferase expression which is controlled by the target sequence of tRF5-GlnCTG. In summary, this is the very first demonstration of the regulatory functions of ELAC2 in the tRF5 induction.

## Methods

### Cell lines, virus, and antibodies

A549 (human alveolar type II-like epithelial), HEp-2 (human epithelial type 2), and 293 (human embryonic kidney epithelial) cells were from ATCC (Manassas, VA) and maintained as described previously (Deng et al., 2015; Choi et al., 2018). Primary cultured human SAE (small airway epithelial) cells were purchased from Lonza (Basel, Switzerland). RSV long strain was grown in HEp-2 cells and purified by sucrose gradient as described previously (Ren et al., 2011; Choi et al., 2018). Viral titer was determined by immunostaining in HEp-2 cells using polyclonal biotin-conjugated goat anti-RSV antibody (7950-0104; Bio-Rad, Hercules, CA) and streptavidin peroxidase polymer (S2438; Sigma-Aldrich, St. Louis, MO), as described previously (Deng et al., 2015; Choi et al., 2018). The antibodies were purchased from the following manufacturers: ANG (sc-74528; Santa Cruz Biotechnology, Santa Cruz, CA), Drosha (sc-393591; Santa Cruz Biotechnology), and Lamin B1 (sc-374015; Santa Cruz Biotechnology), Horseradish-coupled secondary antibodies (sc-2031 and sc-2030; Santa Cruz Biotechnology), β-actin (A1978; Sigma-Aldrich), GAPDH (G9545; Biotrend chemicals, Köln, Germany), Dicer (5362T; Cell Signaling Technology, Danvers, MA), SDHA (5839; Neobiolab, Woburn, MA), RNase L (MBS8292747; MyBioSource, San Diego, CA), ELAC2 (A304-775A-T; Bethyl Laboratories, Montgomery, TX), and V5 (MCA1360GA; Bio-rad).

### Northern blot (NB)

Cellular RNAs were extracted using TRIzol Reagents (Thermo Fisher Scientific, Waltham, MA) and followed by Northern hybridization to detect tRF5-GlnCTG, as described previously (Wang et al., 2013; Zhou et al., 2017a). Briefly, RNA was separated in a 15% denaturing polyacrylamide gel with 7M urea and then transferred to a positively charged nylon membrane (Amersham Biosciences, Piscataway, NJ). The membrane was hybridized with 32P-labeled probes (please see probe sequences in Figure 2A and Supplementary Table S1) in ULTRAhyb-Oligo solution (Life Technologies, Grand Island, NY), followed by washing according to the manufacturer’s instructions.

### Quantitative real-time PCR (qRT-PCR)

qRT-PCR quantification of tRF5-GlnCTG was developed as described with some modifications (Zhou et al., 2017b). In brief, extracted cellular RNA was treated with T4 polynucleotide kinase (T4PNK) to convert the 3’ cyclic phosphate group into a hydroxyl group and ligated to a 3’ RNA linker using T4 RNA ligase, followed by cDNA synthesis. A forward primer specific to tRF5-GlnCTG or RNU6 and a reverse primer specific to a 3’ linker were used to amplify targets. qRT-PCR was performed by using iTaq™ Universal SYBR Green Supermix (Bio-rad) in the CFX Connect Real-Time PCR System (Bio-rad) under the following conditions: initial steps: 50°C, 2 min, and 95°C, 10 min; PCR steps: 95°C, 15 sec and 60 °C, 1 min for 40 cycles. The sequences of primers and a 3’ RNA linker are shown in Figure 2A and Supplementary Table S1.

### Construction of luciferase sensor plasmids and luciferase assays

To identify the functional domain(s) of tRF5-GlnCTG, sensor plasmids containing a complementary sequence of wild-type tRF5-GlnCTG (*Pp*-anti_GlnCTG_WT) or its mutation in 3’-, middle- or 5’- region (*Pp*-anti_GlnCTG_Mut3, *Pp*-anti_GlnCTG_MutM, *Pp*-anti_GlnCTG_Mut5, respectively) in the 3’-untranslated region of the firefly luciferase gene (*Pp*) were constructed, as we described previously (Wang et al., 2013). A sensor plasmid lacking the target site (*Pp*-anti_Control vector) was used as a negative control. All the *Pp* values were normalized by renilla luciferase (*Rr*), another luciferase for internal expression normalization. Briefly, A549 cells were co-transfected with control, WT, or each mutant sensor plasmid and *Rr* for 6 h, followed by mock- or RSV infection. At 24 h post-infection (p.i.), cells were lysed for a luciferase assay using a dual-luciferase kit (Promega, Madison, WI). The relative luciferase activity *(Pp*/*Rr* values) was calculated and compared between WT and mutants.

To investigate the effect of RSV proteins on tRF5-GlnCTG induction, A549 cells were transfected with a plasmid encoding V5-tagged RSV N, P, NS1, or NS2 protein in the presence of a *Pp*-anti_GlnCTG_WT or a *Pp*-anti_Control. *Rr* expression vectors were also co-transfected. At 30 h post-transfection, cells were lysed and followed by luciferase measurement. A plasmid expressing V5 only was used as a negative control for viral protein expression.

### RNA interference

100 nmol/l of siRNA against ANG, RNase L, Dicer, Drosha, or ELAC2 was transfected into A549 cells using Lipofectamine 2000 (Invitrogen). After 40 h, A549 cells were mock- or RSV-infected for 24 h at an MOI of 1, followed by Northern blot (NB), qRT-PCR, or Western blot. The sequence of siRNAs and primers for qRT-PCR is shown in Supplementary Table S1.

### Western blot (WB)

The cell lysates, after protein quantification, were fractionated in SDS-PAGE denaturing gels, as previously described (Choi et al., 2018). The separated proteins were then transferred to polyvinylidene difluoride membranes, followed by incubation with the proper primary antibodies according to the manufacturer’s instructions.

### Subcellular fractionation

Two types of fractionation were performed. One was to isolate nuclei from the total cells, resulting in nuclear and mitochondria-containing cytoplasmic samples, similarly as we described before (Wang et al., 2013). Another one was to prepare the nuclear, mitochondria-free cytoplasmic, and mitochondrial fractions, using the Qproteome Mitochondria Isolation Kit from Qiagen (37612; Valencia, CA), according to manufacturer's instructions. Briefly, A549 cells, mock- or RSV-infected, were washed with 0.9% sodium chloride solution and resuspended in ice-cold lysis buffer. After incubation on ice for 10 min, cell lysates were centrifuged at 1000 x g for 10 min at 4°C and the supernatants were stored as mitochondria-free cytoplasmic fraction. The pellet was resuspended in ice-cold Disruption Buffer and completely disrupted using a syringe with a blunt-ended needle, followed by centrifugation at 1000 x g for 10 min at 4°C. The pellet was stored as the nuclear fraction. The supernatant was further centrifuged at 6000 x g for 10 min at 4°C and the mitochondrial pellet was more purified in the layer of Mitochondria purification and Disruption Buffer. RNA and protein samples of the nuclear, mitochondria-free cytoplasmic, and mitochondrial fractions were prepared for NB and WB, respectively.

### Immunoprecipitation

293 cells were transfected with V5-tagged RSV N, P, or NS1 plasmids. A plasmid expressing V5 only was used as a negative control for viral protein expression. At 30 h post-transfection, cells were lysed and followed by immunoprecipitation using a protein G Immunoprecipitation Kit from Roche (11719386001; Indianapolis, IN), according to manufacturer's instructions, as previously described (Ren et al., 2011; Bao et al., 2013). The anti-V5 antibody was used for the immunoprecipitation and followed by WB to detect endogenously bound ELAC2.

### Statistical analysis

Statistical significance was analyzed using analysis of variance. A *P* value of less than 0.05 was considered significant. Mean±standard error (SE) is shown.

## Results

**Figure 1:**
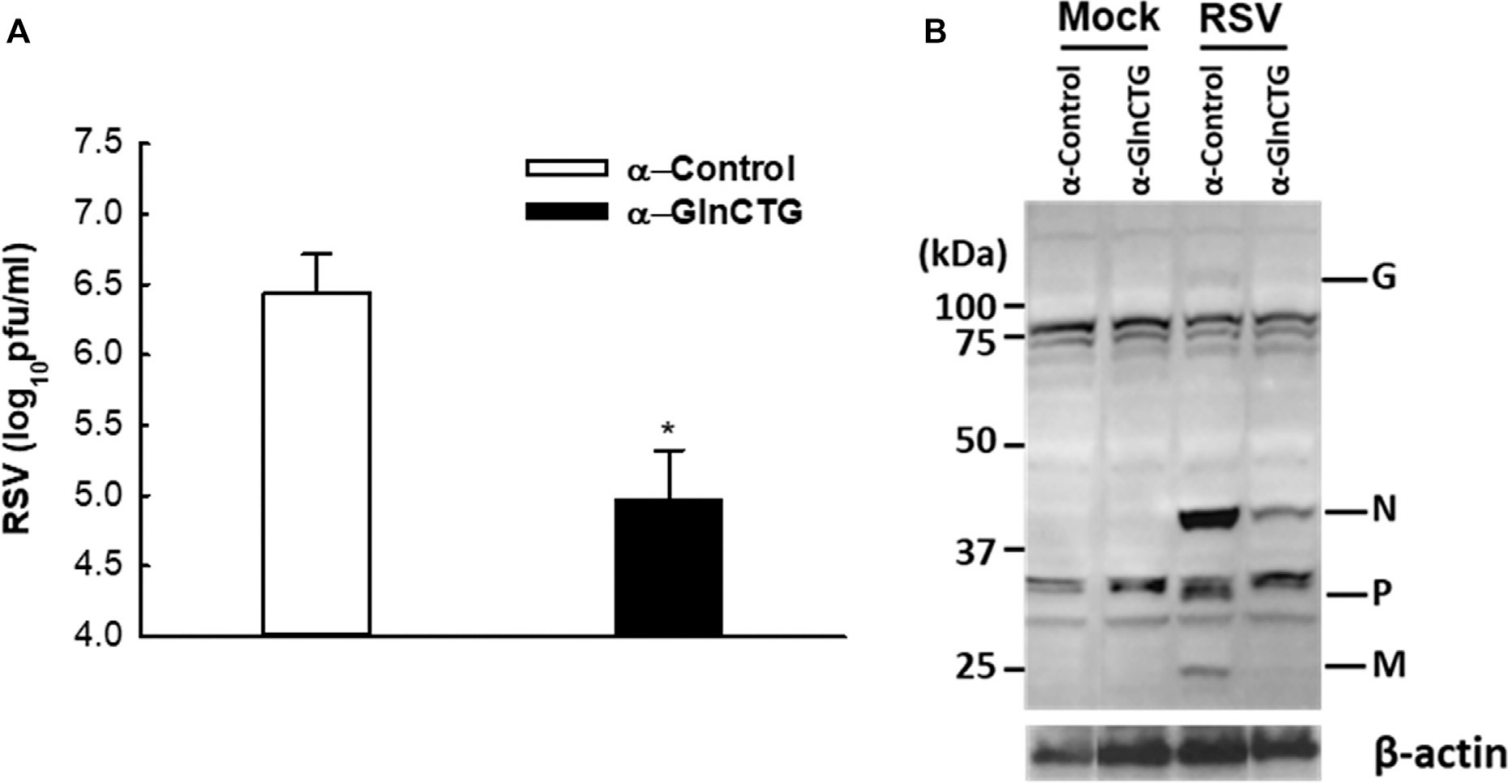
The effect of tRF5-GlnCTG on RSV replication. A549 cells in duplicate were transfected with 120 nmol/l of an antisense oligo against tRF5-GlnCTG (α-GlnCTG) or scrambled oligo (α-Control) using Lipofectamine 2000. At 2 h post-transfection, the cells were mock-infected or infected with RSV at an MOI of 1 for 15 h. **(A)** Total viruses were harvested and infectious particles were quantified by immunostaining using an anti-RSV antibody. **(B)** Total proteins were prepared, and the expression of viral proteins was measured by Western blot using an anti-RSV antibody. RSV proteins are indicated on the right and molecular size markers are on the left. β-actin was used as a control for equal loading of the samples. The sequence of α-GlnCTG and α-Control is shown in Supplementary Table S1. Data are representative of three independent experiments. *P < 0.05, relative to the white bar.

Based on our sncRNA sequencing data (Wang et al., 2013), tRF5-GlnCTG is among the few tRFs whose relative sequencing frequency is more than 10‰ in RSV-infected samples. The sequencing frequency of tRF5-GlnCTG was 0.060‰ and 11.130‰ in mock- and RSV-infected cells respectively, showing 185-fold induction by RSV (Wang et al., 2013). Intriguingly, the fragments of tRNA-Gln-CTG are also inducible by the hepatitis C virus (Jackowiak et al., 2017). Herein, we first tested whether tRF5-GlnCTG regulates RSV replication. For this, we used an antisense oligonucleotide specifically against tRF5-GlnCTG (α-GlnCTG) to inhibit tRF5-GlnCTG or scrambled control oligo (α-control). As shown in Figure 1A, the α-GlnCTG treatment led to a significant decrease in RSV titer, by one and a half log, compared to the α-control treatment. Compared with other RSV-induced tRF5s (Wang et al., 2013; Zhou et al., 2017a), the potent of tRF5-GlnCTG in promoting RSV replication was the highest. Consistent with the suppressive effect of α-GlnCTG on viral replication, WB also showed a decrease in RSV protein expression (Figure 1B), supporting that RSV-induced tRF5-GlnCTG is functional and favors RSV replication.

**Figure 2:**
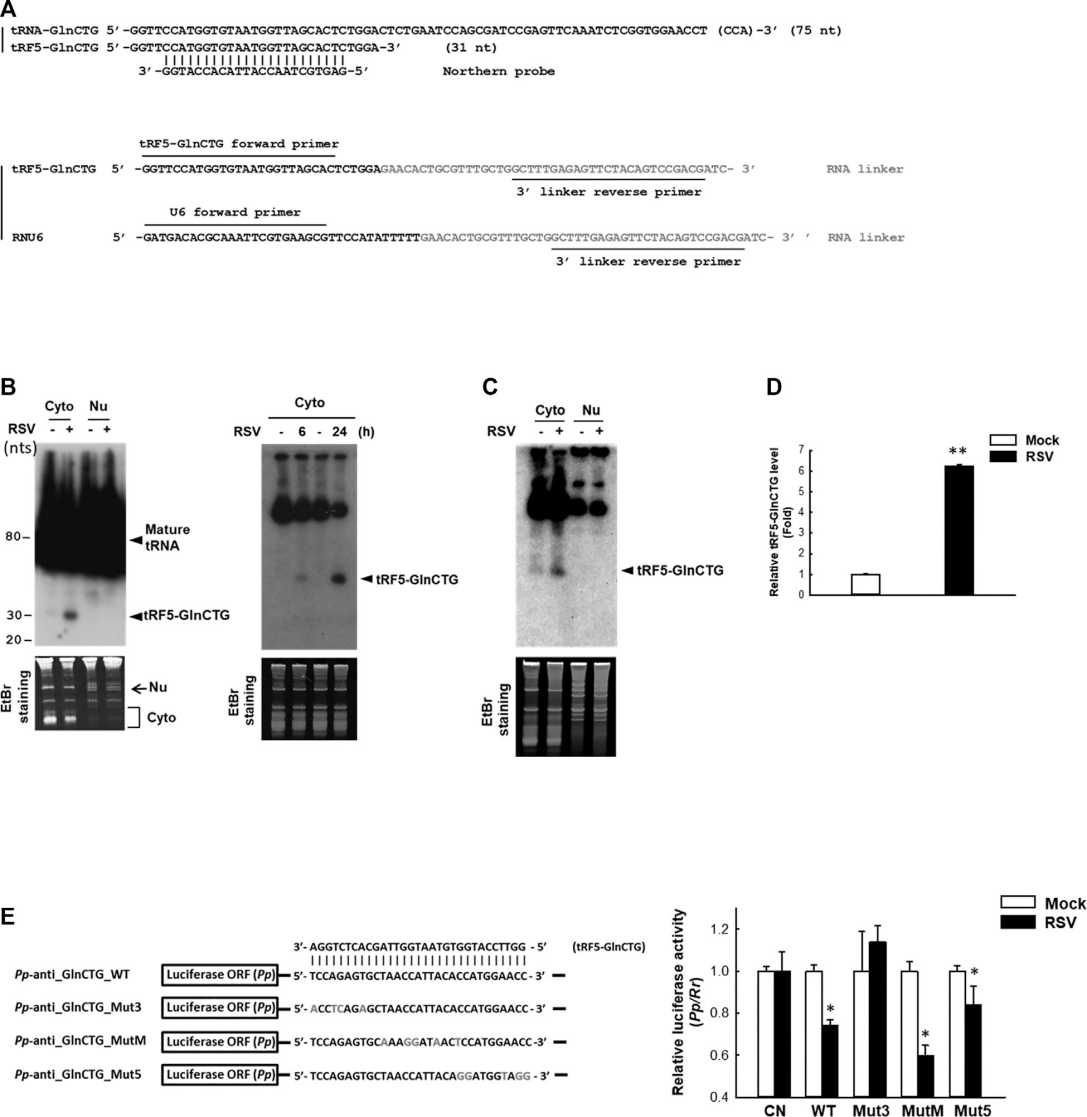
Experimental validation and characterization of tRF5-GlnCTG. **(A)** Sequence alignment of tRF5-GlnCTG with its parental mature tRNA and Northern probe (upper panel), and the illustration of primers and the 3’ RNA linker for tRF detection by qRT-PCR (lower panel). **(B)** A549 cells were mock- or RSV-infected at an MOI of 1. Cytoplasmic (Cyto) and nuclear (Nu) RNAs were purified at 24 h p.i. (left panel) or Cyto RNAs were prepared at 6 and 24 h p.i. (right panel), followed by Northern hybridization to detect tRF5-GlnCTG using the probe indicated in **(A)**. Ethidium bromide (EtBr) staining is shown for equal loading. The size of tRF5 and mature tRNA is indicated on the right; molecular size markers are indicated on the left. Data are representative of 2–3 independent experiments. **(C)** SAE cells were mock- or RSV-infected at an MOI of 1 for 15 h, followed by Cyto and Nu RNA preparation and then Northern hybridization to detect tRF5-GlnCTG using the probe indicated in **(A)**. EtBr staining is shown for equal loading. Data are representative of three independent experiments. **(D)** A549 cells in triplicate were mock-infected or infected with RSV at an MOI of 1 for 24 h, followed by Cyto RNA preparation and then qRT-PCR for the detection of tRF5-GlnCTG. The tRF5-GlnCTG value was normalized to that of RNU6. The sequence of forward and reverse primers are indicated in **(A)** and Supplementary Table S1. Data are representative of three independent experiments. **P < 0.01, relative to the white bar. **(E)** Left panel: Sequence alignment of tRF5-GlnCTG with a sensor plasmid containing targeting sequence of tRF5-GlnCTG (*Pp*-anti_GlnCTG_WT) and three mutant luciferase sensor plasmids (Mut3, MutM, and Mut5). Mutated nts are in red. Right panel: A549 cells in hexaplicate were co-transfected with indicated *Pp* luciferase sensor plasmids and internal control renilla luciferase plasmids (*Rr*). A sensor plasmid lacking the target site (*Pp*-anti_Control vector or CN) was used as a negative control. At 6 h post-transfection, cells were mock- or RSV-infected at an MOI of 1 and then harvested at 24 h p.i. to measure luciferase activities. The relative luciferase activity (*Pp*/*Rr* values) was calculated. Data are representative of three independent experiments. *P < 0.05, relative to the paired white bar.

The subcellular location of a regulatory ncRNA provides an important clue to its function. For example, if an ncRNA has gene regulatory function, its nuclear presence would suggest that it regulates gene(s) at the transcriptional level; while a cytoplasmic ncRNA likely controls gene(s) at the post-transcriptional level. Herein, we investigated the expression and cellular distribution of tRF5-GlnCTG by NB using its specific probe shown in Figure 2A and Supplementary Table S1. We found that tRF5-GlnCTG was exclusively present in the cytoplasm, but not in the nuclei (Figure 2B, left panel), which is consistent with other RSV-induced tRF5s. We also found the induction of tRF5-GlnCTG was time-dependent, as the induction of tRF5-GlnCTG occurred as early as 6 h p.i. and was further enhanced at 24 h p.i. (Figure 2B, right panel). According to our sequencing data, the reading frequency of tRF5-GlnCTG in RSV-infected cells is much lower than that of tRF5-GluCTC, tRF5-GlyCCC, tRF5-LysCTT, and tRF5-CysGCA (Wang et al., 2013). Consistently, the NB signal development for cytoplasmic tRF5-GlnCTG took at least 3 days longer than the signal development for these four tRF5s (data not shown). The induction of tRF5-GlnCTG by RSV was also confirmed in primary cultured human small AEC (SAEC), suggesting the physiological relevance of the induction (Figure 2C). qRT-PCR is a more sensitive way of detection, but a standard protocol seems impractical for tRF detection because PCR primers will also amplify the signal from the corresponding mature tRNAs. To get rid of the signals of tRNAs, qRT-PCR quantification of tRFs was developed as described with some modifications (Zhou et al., 2017b). The modified method did not use the probe-based qRT-PCR, although a probe crossing the end of tRF and the beginning of the RNA linker was proposed to be a key to differentiate the tRF from its corresponding tRNA. However, we found that the linker was enough to differentiate the tRF from its parent tRNA, possibly because the 3’-end of tRNA is always attached with an amino acid preventing the RNA linker attachment. In addition, mature tRNA is in a cloverleaf structure, whose primer annealing requires a specific temperature (Honda and Kirino, 2015; Garcia et al., 2019). Our melt curve of qRT-PCR indeed showed a single peak (Supplementary Figure S1A), suggesting a single PCR product. The loading of PCR product in the denaturing polyacrylamide gel also revealed only a single band around 73 nts (tRF+linker), while there is no band around 117 nts (tRNA+linker), supporting the SYBR Green-based qRT-PCR is suitable for the detection of tRF (Supplementary Figure S1B). We also cloned the qRT-PCR products into the TOPO cloning vector (Invitrogen), and our sequencing data confirmed the presence of tRF only (data not shown). Our qRT-PCR confirmed the induction of tRF5-GlnCTG by RSV infection (Figure 2D).

Our previous studies have revealed that the gene *trans*-silencing by binding to target mRNAs is a common mechanism among several RSV-induced tRFs (Wang et al., 2013; Zhou et al., 2017a). We continued exploring the functions of tRF5-GlnCTG by investigating whether it also has gene *trans*-silencing activity. As shown in Figure 2E, sensor plasmids containing a complementary sequence of wild-type tRF5-GlnCTG (*Pp*-anti_GlnCTG_WT) had a significantly less luciferase expression after RSV infection, while the luciferase expression of a sensor plasmid lacking the target site (CN) was not affected by RSV infection, suggesting the possible interaction between RSV-induced tRF5-GlnCTG with its complementary sequence from the sensor plasmids. We also constructed sensor mutants in 3’-, middle-, or 5’- region (*Pp*-anti_GlnCTG_Mut3, *Pp*-anti_GlnCTG_MutM, and *Pp*-anti_GlnCTG_Mut5, respectively) to confirm the targeting specificity of tRF5-GlnCTG and to identify the region(s) important for the interaction (Figure 2E). The luciferase expression of tRF5-GlnCTG_MutM and tRF5-GlnCTG_Mut5 was sensitive to RSV infection, whereas tRF5-GlnCTG_Mut3 showed unresponsive, indicating that the 3’-portion of tRF5-GlnCTG is important for its gene *trans*-silencing activity, which is distinct from the sequence requirement for miRNA/siRNA. Thus, we found that tRF5-GlnCTG carries out the gene *trans*-silencing function via its 3’-portion.

**Figure 3:**
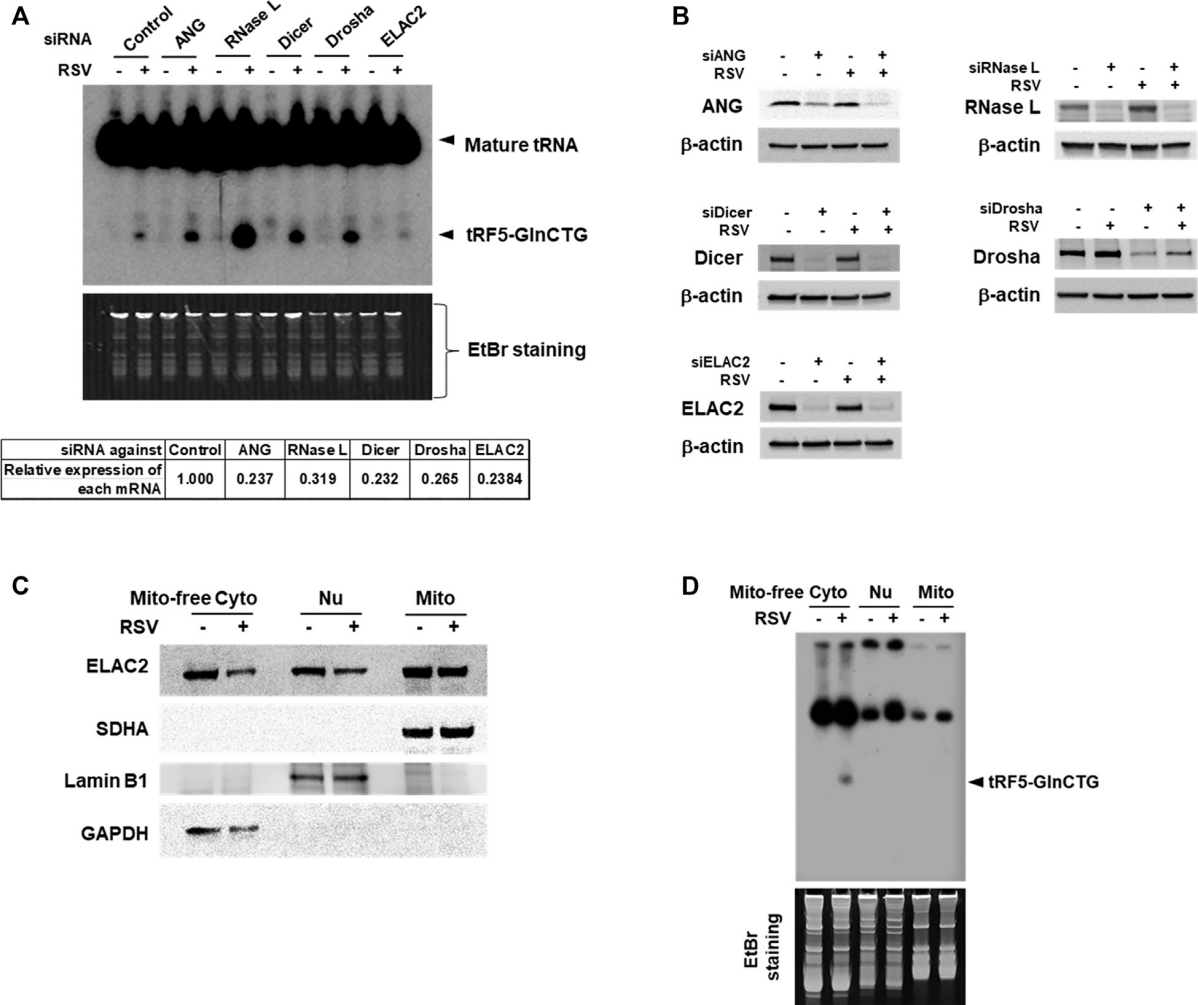
ELAC2 is responsible for the induction of tRF5-GlnCTG. **(A)** A549 cells were transfected with 100 nmol/l of siRNA against individual indicated nuclease or control siRNA. At 40 h post-transfection, the cells were mock- or RSV-infected at an MOI of 1 for 24 h, followed by Cyto RNA preparation and Northern hybridization for the detection of tRF5-GlnCTG. The individual gene knockdown was verified by qRT-PCR (down panel). **(B)** Cells were transfected with siRNAs and then mock- or RSV-infected as described in **(A)**, followed by total protein preparation and then Western blot using antibodies against individual targets. β-actin was used as a loading control. **(C)** Compartmental locations of ELAC2. Cells were uninfected or infected with RSV at an MOI of 1 for 24 h, followed by subcellular fractionation preparation for mitochondria-free cytoplasm (Mito-free Cyto), nuclei (Nu), and mitochondria (Mito) and then Western blot using the indicated antibodies. The purity of fractions was assessed by compartment-specific proteins. The succinate dehydrogenase complex subunit A (SDHA), Lamin B1, and Glyceraldehyde 3-phosphate dehydrogenase (GAPDH) serve as a mitochondrial, nuclear, and mitochondria-free cytoplasmic marker, respectively. Data are representative of 2-3 independent experiments. **(D)** RNA was prepared from subcellular fractionated compartments as described in **(C)**. The same amount of 10 µg in all fractionated RNAs was loaded to a denaturing polyacrylamide gel for Northern hybridization to detect tRF5-GlnCTG. The loading ratio of each fraction was: all Mito RNAs vs 1/25 for Nu RNA vs 1/100 for Mito-free Cyto RNA. Data are representative of 2-3 independent experiments.

Accumulating studies have identified the RNases including ANG, Dicer, and ELAC2 to be involved in tRNA cleavage to generate tRFs (Cole et al., 2009; Rossmanith, 2011; Liu et al., 2018). We previously observed that ANG is responsible for the production of several RSV-induced tRFs (Wang et al., 2013; Zhou et al., 2017a). To explore the induction mechanism of tRF5-GlnCTG, we used siRNAs targeting RNase candidates to investigate whether the induction of tRF5-GlnCTG by RSV gets affected by these candidates. Both ANG and Dicer are involved in the cleavage of some tRNAs, while ELAC2 is well known to be important for tRNA maturation by removing a 3'-trailer from tRNA precursors (Rossmanith, 2011; Siira et al., 2018) or the induction of tRF-1, the tRF derived from the 3’-end of a tRNA precursor transcript that is not retained in the mature tRNAs (Lee et al., 2009). Therefore, we chose these three candidates for the investigation. Other than ANG, Dicer, and ELAC2, we also included Drosha and RNase L for the biogenesis study. It is well known that Drosha is an upstream molecule of Dicer (Bernstein et al., 2001; Zeng et al., 2005), while RNase L is an interferon-induced RNase, cleaving RNA molecules in the cells when it is activated (Liang et al., 2006). As shown in Figure 3A, compared with control siRNA treatment, the depletion of ELAC2 decreased RSV-induced tRF5-GlnCTG. The efficient knockdown of individual RNases by their specific siRNAs was experimentally confirmed (Figures 3A,B). Herein, we found that it was not ANG, but ELAC2 was critical for the biogenesis of tRF5-GlnCTG upon RSV infection.

As mentioned, ELAC2 is an essential enzyme in 3’ tRNA processing for the tRNA maturation in the nuclei and mitochondria (Rossmanith, 2011; Siira et al., 2018). ELAC2 is also responsible for generating cytoplasmic tRF1 which is derived from pre-tRNA-Ser in prostate cancer cells (Lee et al., 2009). The role of ELAC2 in the mitochondria-free cytoplasmic compartment is not clear. To investigate the resource(s) of ELAC2 responsible for the induction of tRF5-GlnCTG by RSV, we performed subcellular fractionation. As shown in Figure 3C, the three compartments (nuclear, mitochondrial, and mitochondria-free cytoplasmic fractionations) were well-purified, as their markers were uniquely present in their respective fraction. We found that while ELAC2 was detected in all fractions, tRF5-GlnCTG was present only in the mitochondria-free cytoplasmic fraction (Figure 3D), supporting that mature tRNA-Gln-CTG, but not pre-tRNA-Gln-CTG, is the target of ELAC2 to generate tRF5-GlnCTG. We also found that the overall expression of ELAC2 was reduced by RSV infection, suggesting that the host may suppress ELAC2 expression to counteract tRF5-GlnCTG induction by RSV, subsequently inhibiting RSV replication and defending viral invasion.

**Figure 4:**
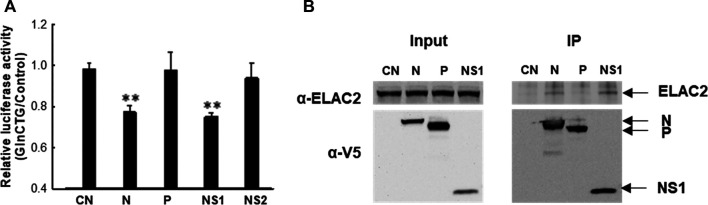
The contribution of RSV proteins to the induction of tRF5-GlnCTG. **(A)** A549 cells in triplicate were transfected with plasmids encoding V5-tagged RSV N, P, NS1, NS2, or empty vector (CN, negative control for viral protein expression). Vectors of *Pp*-anti_GlnCTG_WT (containing the target sequence of tRF5-GlnCTG) or *Pp*-anti_Control (without tRF5-GlnCTG target sequence) were also co-transfected with renilla luciferase plasmids (*Rr*, luciferase expression internal control). At 30 h post-transfection, the cells were harvested for the dual-luciferase assay. *Pp* luciferase values were normalized to their corresponding *Rr* luciferase values and then the normalized value of *Pp*-anti_GlnCTG_WT was normalized to that of *Pp*-anti_Control vector. Data are representative of three independent experiments. **P < 0.01, relative to the first bar. **(B)** 293 cells were transfected with individual V5-tagged viral proteins as indicated. CN and P-expressing plasmids were used as controls. After 30 h, the cells were harvested and immunoprecipitation was done using the anti-V5 antibody, followed by Western blot using the anti-ELAC2 and anti-V5 antibodies. Proper expression of ELAC2 and V5-tagged RSV proteins was confirmed in sample input. Data are representative of three independent experiments.

Similar to many other viruses, RSV employs its viral protein(s) to mediate evasion from antiviral responses. RSV may use its protein(s) to mediate tRF induction to favor viral replication. To test this hypothesis, we expressed several RSV proteins that are not membrane-bound and investigated whether their expression can induce tRF5-GlnCTG by measuring the luciferase expression controlled by the target sequence of tRF5-GlnCTG (*Pp*-GlnCTG) or its control (*Pp*-Control, no target sequence). As shown in Supplementary Figure S2A, both N and NS1 expression significantly suppressed the luciferase expression of *Rr*-corrected *Pp*-GlnCTG. Although NS1 also exhibited some degrees of suppression of *Pp*-Control (Supplementary Figure S2B), the inhibition of NS1 on the expression of *Pp*-GlnCTG was more significant, suggesting a suppression component which is specific against the target sequence. Therefore, we further normalized *Rr*-corrected *Pp*-GlnCTG by *Rr*-corrected *Pp*-Control and showed results in Figure 4A. Overall, the expression of RSV N and NS1 significantly suppressed the luciferase expression controlled by target sequence, while P and NS2 did not, suggesting the role of RSV N and NS1 in the induction of tRF5-GlnCTG. Since ELAC2 is involved in inducing tRF5-GlnCTG, we next examined whether the N and/or NS1 form the complex with ELAC2. For this, we used 293 cells, which are generally used for transfection experiments to investigate protein-protein interaction (Geisse and Fux, 2009; Dalton and Barton, 2014). As shown in Figure 4B, RSV N and NS1 formed a complex with ELAC2 and such formation was RSV replication-independent.

## Discussion

This study demonstrated that RSV-induced tRF5-GlnCTG is functional as it promotes RSV replication. We also showed that ELAC2 is responsible for the induction of tRF5-GlnCTG in RSV infection. The role of ELAC2 in mature RNA cleavage has never been reported. RSV N and NS1 were found to form a complex with ELAC2 and suppressed the luciferase expression controlled by the target sequence of tRF5-GlnCTG, suggesting a possible role of N and NS1 in the induction. We also developed a cost-effective and sensitive method to detect tRF. Since tRF5-GlnCTG regulated the RSV replication, these findings suggest that RSV-induced tRF5-GlnCTG and its responsible RNase could serve as new targets for RSV antiviral drug development.

Currently, multiple methods have been used to detect and quantify sncRNAs including tRFs. NB is the most common method for RNA validation because it can well separate RNAs based on their size and specifically validate the target using a complementary oligo probe (Josefsen and Nielsen, 2011), but with the disadvantages of using hazardous isotope labeling and large sample loading, making the experimental validation difficult if the leftover is limited after the deep sequencing (Streit et al., 2009). In this study, we developed a probe-free qRT-PCR method to validate our deep sequencing data on the induction of tRF5-GlnCTG. The method was convenient and inexpensive, meanwhile, in need of a much less amount of sample with a high detection specificity (Figure 2D and Supplementary Figure S1).

There are three major types of tRFs, namely tRF5s, tRF3s, and tRF1s, which are produced by several RNases. As mentioned, tRF5 is from the 5’-end of its mature tRNA and an endonuclease ANG is responsible for some RSV-induced tRF5s (Wang et al., 2013; Zhou et al., 2017a). ANG has also been demonstrated as responsible for heavy metal- and stress-induced tRF5 and the presence of tRF5s in Hela cells (Sobala and Hutvagner, 2013; Liu et al., 2018). tRF1 is derived from the 3’ end of its tRNA precursor transcript that is not retained in the mature tRNA. In cancer, their biogenesis is controlled by a tRNA 3’-endonuclease ELAC2 (Lee et al., 2009). Dicer, a well-known regulator to produce miRNAs and siRNAs, is also responsible for some tRNA cleavage to produce tRF5s and tRF3s (derived from the 3’-end of mature tRNAs) (Cole et al., 2009; Haussecker et al., 2010; Langenberger et al., 2013). In particular, Dicer is responsible for the biogenesis of tRF5-GlnCTG in Hela cells. However, Dicer-dependent processing of tRNA-Gln-CTG leads to the accumulation of tRF-GlnCTG products with 19 bases in length by the cleavage on D-arm of mature tRNAs (Cole et al., 2009), while RSV-induced tRF5-GlnCTG is 31-nt long (the anticodon loop cleaved product). The role of RNase L in the tRNA cleavage has not been explored until a recent report elucidating that RNase L cleaves tRNA during dsRNA response, with the preference to cleave cytosolic tRNA-His, tRNA-Pro, and tRNA-Gln (Donovan et al., 2017). In Figure 3A, we found that RSV-induced tRF5-GlnCTG was indeed enhanced by RNase L knockdown, suggesting an impossible role of RNase L in cleaving tRNA in the context of RSV infection. These results also suggested that RNase L-mediated tRNA cleavage is stimulus-dependent. Currently, we do not understand why the suppression of RNase L leads to enhanced induction of tRF5-GlnCTG. Is RNase L able to degrade the tRF5-GlnCTG? Is this a new defense mechanism by which the host uses to inhibit RSV replication? If this is the case, how is RNase L activated by RSV? The activation of RNase L in the context of RSV infection is probably not through the interferon (IFN) signaling, as RSV is a poor inducer of IFN in AEC (Bao et al., 2010). Exploring molecular mechanisms used by RNase L to regulate tRF5-GlnCTG induction could be a very interesting study.

In this study, we identified ELAC2 as a novel RNase for the induction of tRF5-GlnCTG by RSV. We also investigated which compartmental ELAC2 is responsible for the tRF induction. As shown in Figure 3C, ELAC2 was present in the nuclei, mitochondria, and mitochondria-free cytosol in A549 cells. However, the RSV-induced tRF5-GlnCTG was only detectable in the mitochondria-free cytoplasmic fraction (Figure 3D), suggesting that the cytosolic ELAC2 is responsible for the tRF induction. The cytoplasmic presence of tRF5-GlnCTG also suggests that the gene *trans*-silencing function of tRF5-GlnCTG, shown in Figure 2E, is at the post-transcriptional level. The gene *trans*-silencing function seems common in many RSV-induced tRF5s. Besides using an artificial luciferase report system, we have previously used a tRF pulldown assay, followed by the RNA high-throughput sequencing, computational analyses, and biological confirmatory experiments, to identify endogenous targets of tRF5-GluCTC, also one of the most abundant RSV-induced functional tRFs (Deng et al., 2015). In the future, we will use a similar method to identify the natural targets of tRF5-GlnCTG. In addition to the gene *trans*-silencing function, tRFs are also involved in translation regulation in a variety of ways including binding to small ribosomal subunits and inhibiting the translation initiation complex (Gebetsberger et al., 2017; Lyons et al., 2017; Akiyama et al., 2020). To fully understand the role of RSV-induced tRF5-GlnCTG, we will also investigate whether tRF5-GlnCTG plays a role in translation regulation in the near future.

Recent developed deep sequencing technologies have revealed ncRNAs to be associated with almost every biological process and exhibit distinct expression patterns in disease states, indicating the potential of ncRNAs as biomarkers or therapeutic targets. Many ncRNA-based therapeutics are currently in clinical applications or clinical trials. For example, onpattro (patisiran, Alnylam Pharmaceuticals) was the first siRNA drug, which was approved for clinical use to treat patients with hereditary transthyretin-mediated (hATTR) amyloidosis by the US Food and Drug Administration (FDA) in 2018 (Kristen et al., 2019). Several miRNAs are also under interventional phase I or II clinical trials to treat liver cancer, lymphoma, melanoma, heart failure, lung cancer, chronic hepatitis C, diabetic non-alcoholic steatohepatitis, and Hereditary nephritis et al., (Hanna et al., 2019; Bajan and Hutvagner, 2020). As mentioned, accumulating evidence supports tRFs to be involved in several human diseases such as cancer and neurodegenerative disease (Selitsky et al., 2015; Li et al., 2019; Zhu et al., 2020). For example, the tRF derived from tRNA-ValAAC is decreased in clear-cell renal cell carcinoma (ccRCC) and correlated with tumor progression (Nientiedt et al., 2016). tRFs produced from tRNA-Glu-YTC, tRNA-Asp-GTC, tRNA-Gly-TCC, and tRNA-Tyr-GTA suppress cancer cell invasion and metastasis (Goodarzi et al., 2015). Furthermore, tRFs were suggested to be involved in the regulation of Parkinson's Disease (PD) progression (Magee et al., 2019). All these reports support that tRFs also have great potential to serve as biomarkers and therapeutic targets in human diseases, similar to other ncRNAs. Our results on the tRF5-GlnCTG and its induction by ELAC2, therefore, may provide novel insight into the development of therapeutic strategies against RSV infection.

## Data Availability Statement

The raw data supporting the conclusions of this article will be made available by the authors, without undue reservation.

## Author Contributions

E-JC and XB contributed to experimental design and data analysis, and wrote the manuscript; WW prepared RSV; E-JC. and KZ performed the experiments; IL, IK, YSL, and XB. contributed to data analysis and interpretation; and XB is responsible for project oversight.

## Funding

This work was supported by grants from the NIH R01 AI116812 and R21 AG069226, and FAMRI Clinical Innovator Award 160020 to XB.

## Conflict of Interest

The authors declare that the research was conducted in the absence of any commercial or financial relationships that could be construed as a potential conflict of interest.
