# Supplementary material for: ELAC2, an Enzyme for tRNA Maturation, Plays a Role in the Cleavage of a Mature tRNA to Produce a tRNA-Derived RNA Fragment During Respiratory Syncytial Virus Infection
Source: Front Mol Biosci. 2021 Feb 2;7:609732. doi: 10.3389/fmolb.2020.609732 (PMC7884774; doi:10.3389/fmolb.2020.609732)
Supplement: Supplementary file 1 [file datasheet1.pdf]

**Supplementary Table S1. Sequence of antisense oligos, a Northern probe, Primers, and siRNAs used in this study**

| Name | Sequence (5' to 3') |
|---|---|
| **Antisense oligos** | |
| anti-Control | [mC][mC][mG][mC][mU]GAGCTAAAGC[mC][mA][mG][mC][mC] |
| anti-GlnCTG | [mG][mA][mG][mU][mG]CTAACCATTACAC[mC][mA][mU][mG][mG] |
| **A Northern probe** | |
| tRF5-GlnCTG probe | GAGTGCTAACCATTACACCATGG |
| **Primers for qRT-PCR** | |
| tRF5-GlnCTG forward | GGTTCCATGGTGTAATGGTTAG |
| RNU6 forwad | GATGACACGCAAATTCGTGAAGCG |
| 3' linker reverse | CGTCGGACTGTAGAACTCTCAAAGC |
| 3' RNA linker | [Phos]GAACACUGCGUUUGCUGGCUUUGAGAGUUCUACAGUCCGACGAUC-[23ddC] |
| ANG forward | TCTGAGGCCGAGGAGCC |
| ANG reverse | GTGTACCTGGAGTTATCCTGAGCC |
| RNase L forward | TGAGTGACCTGCTGGGTCATC |
| RNase L reverse | TGCAGTAGTCTGAGGATCTCACTTTC |
| Dicer forward | CATGAGGGCCGCCTTTC |
| Dicer reverse | CACCATGCGGCTGGGTAG |
| Drosha forward | CCCATGCCCGAACCTACAC |
| Drosha reverse | CAAGCGCATCCATTGCTG |
| ELAC2 forward | TGAACGCGGAGTTCATTATGC |
| ELAC2 reverse | TTGTTGGAAAGTCTCCAAAGCAG |
| 18S forward | ACATCCAAGGAAGGCAGCAG |
| 18S reverse | TCGTCACTACCTCCCCGG |
| **siRNAs** | |
| Control | GCGCGAUAGCGCGAAUAUA[dT][dT] |
| ANG-1 | GACAUCAACACAUUUAUUC[dT][dT] |
| ANG-2 | CACAUUUAUUCAUGGCAAC[dT][dT] |
| RNase L | GAAGAUGAAUUUGCCCGAA[dT][dT] |
| Dicer | Stealth siRNAs (HSS118717, HSS118718, HSS118719) from Invitrogen |
| Drosha | Stealth siRNAs (HSS120887, HSS178991, HSS178992) from Invitrogen |
| ELAC2 | GUGUCCGAGACUUAACGAA[dT][dT] |
