# Supplementary figures and images for: ELAC2, an Enzyme for tRNA Maturation, Plays a Role in the Cleavage of a Mature tRNA to Produce a tRNA-Derived RNA Fragment During Respiratory Syncytial Virus Infection
Source: Front Mol Biosci. 2021 Feb 2;7:609732. doi: 10.3389/fmolb.2020.609732 (PMC7884774; doi:10.3389/fmolb.2020.609732)

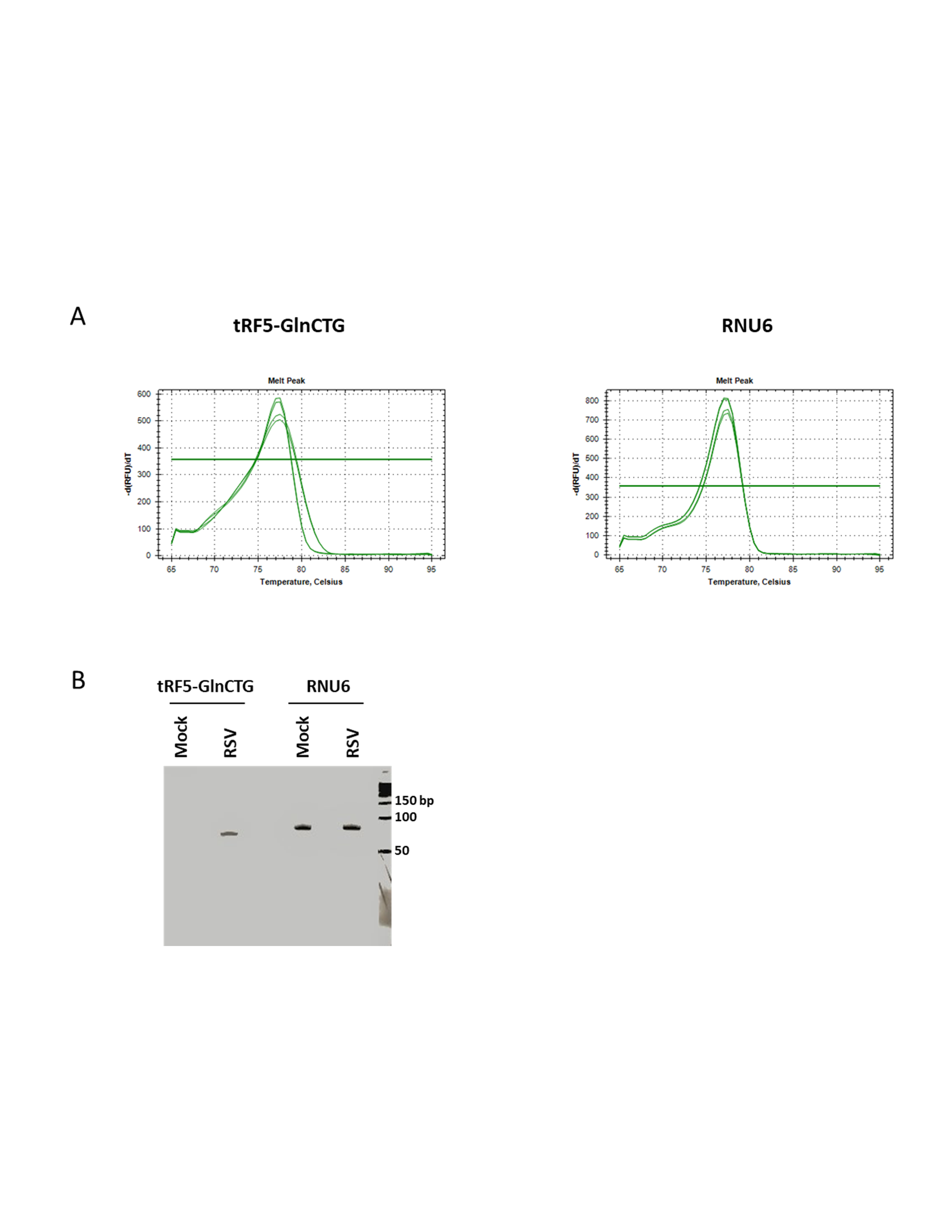

Supplement: Supplementary file 2 [file image1.tif]

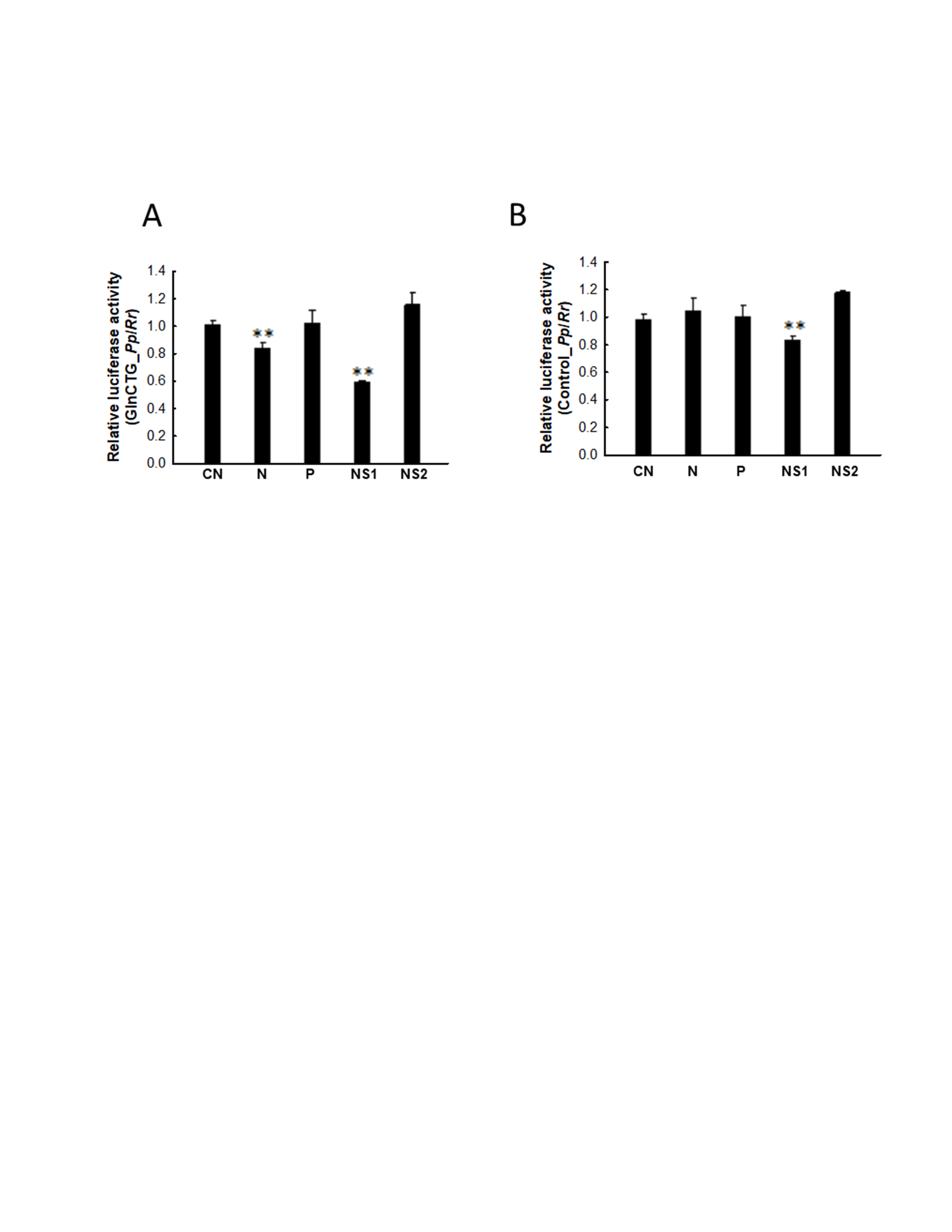

Supplement: Supplementary file 3 [file image2.tif]
